# Prince Tafari at St. Thomas's Hospital

**Published:** 1924-08

**Authors:** 


### PRINCE TAFARI VISITS ST. THOMAS'S HOSPITAL.

During the stay in London of H.I.H. Tafari Makonnen, heir to the throne of Abyssinia and, it is claimed, a descendant of the Queen of Sheba and King Solomon, St. Thomas's Hospital was honoured with a royal visit. Whilst the arrangements for his stay in England were being made the Prince expressed a wish to visit St. Thomas's Hospital in order to see the panels in tiles, illustrating the stories of Little Jack Horner and Old King Cole, of which he had been told by his father who gave them to the hospital twenty years ago. The Prince with his suite was met at the hospital by Sir Arthur Stanley, the treasurer, Mr. G. Q. Roberts, the secretary, who had taken round the late Emperor of Abyssinia on the occasion of his visit, and Miss Still, the matron. They visited first the out-patients' department and two wards for adults, then they passed on to the children's ward where the nursery-rhyme panels are. When told, on inquiring, that the panels had cost his father £100 he said that, as money value is so much less now, he would give £300 This will be used to build a balcony outside one of the children's wards.

As the Prince wished to see the operating theatre, he was taken there next and presented to the senior surgeon, Sir Cuthbert Wallace, who was at that time performing an operation for the removal of glands from a patient's neck. The Prince showed an eager interest in the operation and wished to see one from the beginning, but his suite, with handkerchiefs to their noses, found the smell of the anæsthetic so overpowering that one of them had to be taken out lest he should faint. From the theatre the visitors went on to one of the orthopædic wards where a number of patients were receiving sun treatment While there he asked to be taken to see the disease and germ specimens preserved in spirits, and the morning ended with a visit to the museum of surgical pathology. Before leaving a ward H.I.H. always asked the sister-in-charge to express his sympathy to each patient. As he drove away he was loudly cheered by a party of students, an acclamation which appeared to please him greatly.

